# Aortopexy for the treatment of tracheomalacia in children: review of the literature

**DOI:** 10.1186/1824-7288-38-62

**Published:** 2012-10-30

**Authors:** Michele Torre, Marcello Carlucci, Simone Speggiorin, Martin J Elliott

**Affiliations:** 1 Paediatric Surgery, G. Gaslini Institute, Genova, 16145, Italy; 2 The National Service for Severe tracheal Disease in Children, The Great Ormond Street Hospital for Children NHS Trust, London, WC1N 3JH, United Kingdom

**Keywords:** Tracheomalacia, Aortopexy, Sternotomy, Thoracotomy, Thoracoscopy, Bronchoscopy, ALTE, Gastro-oesophageal reflux, Tracheal stent, Oesophageal atresia

## Abstract

**Abstract:**

Severe tracheomalacia presents a significant challenge for Paediatricians, Intensivists, Respiratory Physicians, Otolaryngologists and Paediatric Surgeons. The treatment of tracheomacia remains controversial, but aortopexy is considered by most to be one of the best options. We conducted a review of the English literature relating to aortopexy.

Among 125 papers, 40 have been included in this review. Among 758 patients (62% males) affected with tracheomalacia, 581 underwent aortopexy. Associated co-morbidities were reported in 659 patients. The most frequent association was with oesophageal atresia (44%), vascular ring or large vessel anomalies (18%) and innominate artery compression (16%); in 9% tracheomalacia was idiopathic. The symptoms reported were various, but the most important indication for aortopexy was an acute life-threatening event (ALTE), observed in 43% of patients. The main preoperative investigation was bronchoscopy. Surgical approach was through a left anterior thoracotomy in 72% of patients, while median approach was chosen in 14% and in 1.3% a thoracoscopic aortopexy was performed. At follow-up (median 47 months) more than 80% of the patients improved significantly, but 8% showed no improvement, 4% had a worsening of their symptoms and 6% died. Complications were observed in 15% of patients, in 1% a redo aortopexy was deemed necessary.

In our review, we found a lack of general consensus about symptom description and evaluation, indications for surgery, though ALTE and bronchoscopy were considered by all an absolute indication to aortopexy and the gold standard for the diagnosis of tracheomalacia, respectively. Differences were reported also in surgical approaches and technical details, so that the same term “aortopexy” was used to describe different types of procedures. Whatever approach or technique was used, the efficacy of aortopexy was reported as high in the majority of cases (more than 80%). A subgroup of patients particularly delicate is represented by those with associated gastro-esophageal reflux, in whom a fundoplication should be performed. Other treatments of tracheomalacia, particularly tracheal stenting, were associated with a higher rate of failure, severe morbidity and mortality.

**Non english abstract:**

La tracheomalacia severa rappresenta una sfida per Pediatri, Intensivisti, Pneumologi, Otorinolaringoiatri, Chirurghi Pediatri. Il trattamento della tracheomalacia è tuttora controverso. L’aortopessi è considerata da molti la migliore opzione terapeutica. Abbiamo condotto una revisione della letteratura di lingua inglese su tale argomento.

Di 125 lavori, 40 sono stati inclusi nella revisione. Tra 758 pazienti (62% maschi) affetti da tracheomalacia, 581 sono stati sottoposti ad aortopessi tra il 1968 e il 2008. In 659 pazienti alcune comorbidità erano presenti. L’associazione più frequente era con l’atresia esofagea (44%), l’anello vascolare o un’anomalia dei grossi vasi (18%), la compressione da parte dell’arteria innominata (16%); nel 9% la tracheomalacia era idiopatica. I sintomi riportati sono stati variabili, ma l’indicazione più importante all’aortopessi sono stati eventi di ALTE, osservati nel 43% dei pazienti. Lo studio diagnostico preoperatorio principale è stato la broncoscopia. L’approccio chirurgico è avvenuto attraverso una toracotomia anteriore sinistra nel 72% dei pazienti, mentre un approccio mediano è stato scelto nel 14% e nell’1.3% dei casi è stato eseguito un approccio toracoscopico. Al follow-up (mediana di 47 mesi) la maggioranza dei pazienti sono migliorati significativamente, ma l’8% di essi non è migliorato, il 4% è peggiorato e il 6% è morto. Complicazioni sono state riportate nel 15% dei pazienti, nell’1% un nuovo intervento di aortopessi è stato necessario.

In questa revisione abbiamo trovato che non c’è un consenso generale sulla valutazione e sulla descrizione dei sintomi, sulle indicazioni chirurgiche ed esami preoperatori, anche se le ALTE e la broncoscopia venivano considerate rispettivamente un’indicazione assoluta all’aortopessi e il “gold standard” diagnostico per la tracheomalacia. Venivano riportate differenze negli approcci chirurgici e nei dettagli tecnici, e lo stesso termine di aortopessi veniva usato per indicare diverse procedure chirurgiche. In ogni caso, indipendentemente dall’approccio o tecnica utilizzati, l’efficacia dell’aortopessi veniva riportata come elevata nella maggioranza dei casi (più dell’80%). Un sottogruppo di pazienti particolarmente delicato è rappresentato da quelli con reflusso gastroesofageo associato, nei quali sarebbe indicata una fundoplicatio. Altri trattamenti della tracheomalacia, quali stent tracheale, sembrano gravati da una maggiore percentuale di insuccessi, morbidità severa e mortalità.

## Introduction

The aim of this paper is to evaluate and discuss the literature relating to indications, surgical details and clinical results of aortopexy, usually performed for tracheomalacia (TM).

TM is a localized or generalized weakness of the tracheal wall which creates airway obstruction resulting in different degrees of symptoms. It can be isolated or associated with other anomalies such as anterior vascular compression, oesophageal atresia (OA) with tracheo-oesophageal fistula (TOF) or gastro-oesophageal reflux (GOR). Although, in some cases, spontaneous improvement can occur, TM can also result in severe cough, respiratory distress episodes or “near-death” spells (acute life-threatening events, ALTE). Amongst several possible treatments, including tracheostomy and non invasive ventilation, airway stenting, and surgical approaches, aortopexy is a favoured option in many centres. Aortopexy means lifting anteriorly the aorta and suturing it to the posterior surface of the sternum. As the anterior tracheal wall is attached through pre-tracheal fascia to the posterior aortic wall, the tracheal lumen is opened by aortopexy.

Despite this popularity, there is a surprising lack of evidence to support aortopexy as effective treatment for TM [1], and no randomized controlled trials have been published on this subject. Most papers report only small, single centre series.

Moreover, the term ‘aortopexy’ is rather generic and may describe many different approaches and different techniques. The approach to the aorta can be anterior, through a median full or limited sternotomy [2], possibly associated with cervical incision, lateral or anterolateral [3], from both sides of the thorax. More recently, thoracoscopic aortopexy has been described [4].

## Materials and methods

A literature review was conducted on PubMed, using the search term “Aortopexy” without setting any temporal or other limits. Inclusion criteria for the review were papers written in English reporting more than one case of aortopexy. The references articles of the selected papers were screened and included if they met the inclusion criteria.

A total of 125 papers were identified, but only 40 papers met the inclusion criteria and thus form the subject of our review. The articles were classified according to the revised SIGN grading system, available on http://www.sign.ac.uk/guidelines/fulltext/50/section6.html. The following data have been retrieved from every paper included: demographics (number, sex and age of the patients), causes of TM, clinical data (symptoms, co-morbidities), diagnostic investigations, type of treatment (approach to aortopexy, other surgical procedures), outcome (complications, resolution of symptoms and length of follow up).

## Results

Table 1 shows the list of the papers evaluated and the summary of the data retrieved. We analyzed 40 articles. Of these, 14 reported less than 10 patients, 13 papers between 10 and 20 patients and only 16 articles more than 20 patients.

**Table 1:** Aortopexy review of the literature

***Author***	***Period***	***SIGN classification***	***N°***	***Cause of TM***	***Diagnostic***	***Intraoperative broncoscopy***	***Operation***	***Complication/ reoperations***	***Outcome***	***F-UP months***
Schwartz et al. J Pediatr Surg 1980	1977-1979	D	8	OA	8BS 8B	8	8 AP	1 VRD	7G 1D	20
Blair et al. J Pediatr Surg 1986	1978-1985	C	25	18OA 6AAA 1 O	25BS 25B	23	21 AP 2 AP+TS 2 TS	4 TS 1 VRD	21G 4D	48
Greenholz et al. J Pediatr Surg 1986	1977-1984	C	41	5 OA 6 IA 10 AAA 17 IT 6 O	28 BS 27 AG 41B	17	19 AP 1 LTP 11 O 15 NS	-	35 G 2 P 4D	21
Malone et al. Arch Dis Child 1990	1986-1988	C	17	17 IT	17B	-	17 AP	4 AP+TP 4 T	7G 9W 1D	RS
Clevenger et al. Ann Thorac Surg 1990	1985-1990	C	12	6 OA 10 IA 2 AAA	6 BS 12 B	-	12 AP 3 VRD	3 recurrent apnoea 3 ventilator dependence 11 major complication	5 G 6 P 1 D	10.5
Dohlemann et al. J Pediatr Surg 1990	1990	D	3	1 OA 2O	2 BS 2 AG 3 B	3	3 AP	-	3G	-
Brawn et al. J Pediatr Surg 1991	1991	D	2	1 OA 1 PI	1 BS 2B	-	2 AP	1 bleeding 1AP+TP	1G 1W	7
Yamaguchi et al. Eur J Cardiothorac Surg 1991	1978-1990	C	21	3 OA 2 AAA 14 PAA	21 B	17	3 AP 5 LTP 14 PPA	1 AP after LTP	15 G 1 W 1 D	109
Filler et al. J Pediatr Surg 1992	1977-1991	C	32	32 OA	32 B	-	29 AP 2 AP + TS 1 TS	1 seroma 1 vocal chord paralysis 1 stent dislogment 1 LTP 1 lobectomy	28 G 3 W 1 D	6.5
Chun et al. Ann Thor Surg 1992	1968-1990	C	39	39 AAA	39 BS 33 AG	-	10 AP 39 VRD	3 bleeding 2 respiratory arrest 5 BPN 1 PNX 1 chylotorax 1 sepsis	37 G 2 D	12.5
Corbally et al. Eur J Pediatr Surg 1993	1980-1990	C	48	48 OA	48 B	-	48 AP	1 bleeding 1 TP 21 Nissen 1 phrenic nerve palsy	38 G 5 P 2 D	46
Jonas et al. Ann Otol Rhinol Laryngol 1994	1989-1993	D	12	12 IA	12 B	-	12 AP	4 persistent stridor	8 G 4 G	24
Triglia et al. Ann Otol Rhinol Laryngol 1994	1987-1983	C	12	12 OA 12 IA 4 AAA	12 B 3 AG	12	12 AP	1 reimplantation of innominate artery (post-trauma)	9 G 3 W	24
Roberts et al. J Pediatr Surg 1994	1976-1992	C	30	30 AAA	28 BS 28 AG 16 B	-	9 AP 17 VRD	1 AP-TP 2 TP 1 aortoesophageal fistula	26 G 4 P	-
Masaoka et al. Eur J Cardiothorac Surg 1996	1996	D	5	2 OA 1 AAA 2 O	5 B	-	1 AP 1 LTP 1 BR 2 NS	1 persistent TM	3 G 1 P 1 D	-
Bullard et al. J Pediatr Surg 1997	1989-1994	D	6	5 OA 1 AAA	-	-	6 AP	1 PNX 2 hemotorax	4 G 2 P	-
McCarthy et al. Eur J Cardiothorac Surg 1997	1983-1995	C	24	6 IA 9 AAA 2 PAA 5 TBS	9 BS 24 B		6 AP 9 VRD 6 LTP 3 O	1 AP 1 TBR	19 G 2 P 3 D	40
McElhinney et al. Ann Thorac Surg 1999	1993-1997	D	5	5 AAA	1 BS 2 AG 5 B	-	5 AP	2 T	3 G 2 P	24
Gormley et al. J Pediatr Otorhinolaryngol 1999	1986-1998	D	16	15 IA 1AAA	16 BS 16 B 6 AG	-	11 AP 1 VRD 4 NS	-	12 G 3 P 1 W-	-
Kamata et al. J Pediatr Surg 2000	1992-1997	D	14	4 OA 4 IT 6 O	14 B	14	13 AP 1 PPA	-	12 G 2 D	50
Morabito et al. J Pediatr Surg 2000	1978-1999	D	16	15 OA 1 IA	16 B	16	9 AP 4 TP 3 LTP	1 T 1 Nissen	15 G 1 P	-
Dave et al. J Pediatr Surg 2006	1981-2004	B	28	15 OA 13 IT	28 BS 28 B	7	28 AP	1 phrenic nerve palsy 3 lung collapse 1 tymic compression	26 G 2 P	6
Vazquez et al. Ann Thorac Surg 2001	1985-2000	C	29	27 OA 1 IA 1 AAA	14 AG 29 B	-	29 AP	1 phrenic nerve palsy 3 PNX 1 scoliosis	28 G 1 P	8
Weber et al. Am J Surg 2002	-	C	32	18 OA 4 IA 8 AAA 2 IT	32 B	32	32 AP	2 reintubations	26 G 4 P 2 W	6
Schaarschmidt et al. J Pediatr Surg 2002	2000	D	2	1 OA 1 AAA	2B	2	2 AP	-	2 G	17
Ahel et al. Pediatr Int 2003	1994-2001	D	3	1 OA 2 IT	3 B	-	3 AP	-	2 G	-
Valerie et al. J Pediatr Surg 2005	1993-2003	B	25	17 OA 8 O	25 B	-	11 AP 14 TS	1 reintubation 4 pericerdial effusion	-	-
Khatami et al. Thorac Cardiov Surg 2006	1995-2004	D	5	5 OA	5 B	-	5 AP	-	-	-
Van der Zee et al. Surg Endosc 2007	2002-2005	D	6	6 OA	6 B	-	6 AP	2 AP	4 G 2 P	-
Grillo et al. Ann Thorac Surg 2007	-	D	4	4 AAA	3 AG 4 B	4	4 AP 3 VRD	1 hemotorax 2 chylotorax 1 TS	3 G 1 W	108
Abdel-Rahman et al. - World J Surg 2007	1992-2006	C	20	5 OA 2 AAA 13 IT	20 B	20	20 AP	1 AP	16 G 2 P 2 W	13
Perger et al. J Laparosc Adv Surg Techn 2009	2003-2006	D	5	4 OA 1 IT	-	4	5 AP	1 T	3 G 1 P 1 W	26
Fraga et al. J Pediatr Surg 2009	1996-2008	D	4	4 AAA	4 B	4	4 AP	-	3 G 1 P	110
Gardella et al. J Pediatr Surg 2010	1997-2006	B	28	28 IA	28 B	16	16 AP 12 NS	-	16 G	48
Calkoen et al. Pediatr Crit Care Med 2011	1990-2008	B	105	44 OA 16 AAA 45 O	103 B	18	105 AP	4 AP 14 T 5 TS	76 G 24 P	84
Horvath Eur J Cardiothorac Surg 1992	1979-1992	C	26	26 IA	26 B 26 BS	-	26 AP	-	25 G 1 P	46
Kiely et al. Ped Surg Int1987	1980-1986	C	25	22 OA 3 IT	-	-	25 AP	1 hemothorax 1 heart failure 4 infections 2 TP	17G 5P 3W	-
Filler J Pediatr Surg 1976	1974-1975	D	3	3 OA 1 IA	3 BS 3 B	-	3 AP	-	-	-
Vinograd J Cardiovasc Surg 1994	1988-1992	C	20	-	20 B	20	19 AP 1 PPA	3 LTP 1 TS	14 G 4 P 2 W	

**Table legend**: Literature review: period, number and sex of patients, diagnostic, type of surgery and outcome are shown. Articles were classified according to revised SIGN grading system.

List of abbreviations in Table 1:

AAA= Aortic Arch Anomalies.

AG= Angiography.

AP= Aortopexy.

BG= Bronchography.

B= Bronchoscopy.

D= Dead.

G= Good.

BS= Barium Swallow.

IA= Innominate Artery Compression.

IT= Idiopathique Tracheomalacia.

LTP= Laryngotracheoplasty.

NS= Non Surgical Treatment.

O= Other.

OA= Oesophageal Atresia.

P= Poor/No Improvement.

PI= Post Intubation.

PAA= Pulmonary Artery Anomalies.

PPA= Pulmonary Artery Plico-Suspension.

RS= Retrospective Study.

T=Tracheostomy.

TBR= Tracheo-Bronchial Resection.

TBS= Tracheo-Bronchial Stenosis.

TM= Tracheomalacia.

TP= Tracheopexy.

TS= Tracheal Stenting.

VRD= Vascular Ring Division.

W= Worsening.

A total of 758 patients (62% males) were affected with TM; 581 of them underwent aortopexy at a mean age of 10.5 months between 1968 and 2008. TM was associated with OA in 44% of patients; in 18% vascular rings or other anomalies of the heart or great vessels was reported; and in 16% there was right innominate artery compression. In 9% the TM was classified as idiopathic. The most frequent symptom was one or more episodes of ALTE (43%), followed by stridor (26%), recurrent pneumonias (21%), respiratory distress (14%), cough and/or wheezing (8%), dysphagia (4%) or impossibility to wean from mechanical ventilation (3.5%). In a population of patients with TM and innominate artery compression [5], the presenting symptom varied according to age, as small infants presented more frequently with ALTE or apnoeic episodes and older children with cough episodes. Among pre-operative investigations, bronchoscopy was performed in 98% of cases.

The surgical approach was mainly via thoracotomy; left anterior in 72% of cases, right anterior in 9% and muscle sparing in 2%. A median approach (partial or full sternotomy) was electively chosen in 15%. This solution was also adopted instead of another approach if another cardiovascular procedure had to be performed at the same time. In the largest series published [6], left thoracotomy and median sternotomy were compared and no differences have been found in terms of efficacy of the aortopexy. In 1% of patients a thoracoscopic aortopexy was performed. Intra-operative bronchoscopy was performed in 37% of cases to evaluate the resolution of the tracheal collapse during the maneuver, but the majority of the surgeons (58%) do not describe it as necessary. One of them reported using intra-operative bronchoscopy during their initial experience but discarded it later on [7].

The mean and median follow up was 42 and 47 months, respectively. More than 80% of the patients had a satisfactory improvement of the symptoms, 8% did not improve, 4% had a worsening of their symptoms and 6% died. As a lower success rate in patients with GOR was described, [8,9] fundoplication prior [10] or after the aortopexy [7] was suggested. In a study in which the efficacy of aortopexy was compared in different populations [6], good results were observed after aortopexy in patients with TM associated with OA and TOF, vascular compression or primary TM. The only group with poorer outcome was represented by the patients with associated severe cardiac anomalies or other severe comorbidities.

In the study comparing two groups of patients with TM by innominate artery compression, presenting at different ages, the success rate of aortopexy was satisfactory in both groups [5].

Objective improvement of respiratory function after aortopexy, in particular FEV1, was reported by two authors [11,12].

The most frequent complications of aortopexy were: pneumothorax or pleural effusion (3%), lung atelectasis (2.5%), pericardial effusion (2%), phrenic nerve palsy (1.3%), bleeding (1%). Re-do aortopexy was reported in less than 1% of cases. Other surgical procedures performed before, in association with, or after, aortopexy were; vascular ring division (17.5%), external or internal stenting of the trachea (5%), tracheal resection or tracheoplasty (3.7%), and tracheopexy (1.3%) Three studies reported a predisposition of the patients to get chest infections, even long after aortopexy [11,13,14].

## Discussion

The review of the literature showed that TM may be associated with different conditions, such as OA, vascular rings, and innominate artery compression. The difference in TM types and the variety of associations result in data which are difficult to analyze. There are few series reporting experience from a significant number of patients and obvious differences in symptom description, modalities of pre-operative investigations, surgical indications, type of approach, type and modality of evaluation of the results further complicate the analysis.

There is a lack of general consensus about the indication for surgery. In the majority of the series (78%) a history of repeated infections or not well specified “stridor” are included among the indications to aortopexy, while in 22% of the series the only indications were represented by severe apneas or ALTE or failed extubation. However, general agreement does seem to exist between the authors that one or more episodes of ALTE are an indication for aortopexy, though ALTE represents only less than 50% of surgical indications to aortopexy in the series evaluated.

Another matter not well defined is which pre-operative investigations are necessary.

All the following investigations have been reported in the series evaluated to diagnose TM and comorbidities: laringo-tracheo-bronchscopy, oesophago-gastroscopy, barium swallow, angiography, bronchography, lateral chest radiograph, computerized tomography scan, magnetic resonance, oesophageal manometry, ph study, echocardiography. A matter of agreement was the use of endoscopy as the most useful investigation to diagnose TM. Both rigid and flexible bronchoscopy were used and were able to help diagnose TM under spontaneous breathing. While flexible bronchoscopy is less traumatic, the advantage of rigid bronchoscopy is to better evaluate the presence of a proximal or recurring TOF, the length and diameter of the malacia. The percentage of lumen obstruction that represents an indication to aortopexy is controversial, varying, according to the different authors, from 30% to 70% [5,15]. The use of bronchography has been abandoned by most centres, but it is still used in association with bronchoscopy to obtain more information from a combined investigation [6,16]. Bronchography gives a dynamic and morphologic evaluation of whole tracheobronchial tree and allows an accurate measurement of the airway lumen under different ventilation pressures. On the other hand, CT scan is useful to diagnose vascular anomalies [16].

Very different opinions exist between authors on the value of intra-operative bronchoscopy, which is advocated by many as necessary in order to make sure that aortopexy will be effective and not mentioned or considered useless by others [7]. Intra-operative bronchoscopy can be used to determine the number of stitches necessary to improve the tracheal lumen. In our study, intra-operative bronchoscopy was preferably performed in more than one third of cases, while it was considered unnecessary by 58% or only seldom performed in 12.5% of cases.

There are many differences between the authors regarding the surgical approach to aortopexy (Table 2). Left anterior thoracotomy can be considered the preferred approach by the vast majority of surgeons (72% of the patients received a left anterior thoracotomy), but up to one third of the patients are treated with other surgical approaches. Thoracoscopy has been used in 1% of cases, belonging to three centres, though apparently with satisfactory results, [4,13,17]. The theoretical advantage of thoracoscopic aortopexy is obviously to reduce the postoperative pain and the morbidity of a thoracotomy or sternotomy.

**Table 2:** Surgical approach in aortpexy procedure

**Surgical Approach**	**%**
**LAT**	71,94%
**RAT**	8,95%
**MS**	11,88%
**BILATERAL THORACHOTOMY**	0,17%
**MEDIASTINAL APPROACH**	1,03%
**LPT**	1,55%
**MANUBRIUM SPLIT**	2,75%
**LTH**	1,03%
**RTH**	0,17%
**MUSCLE-SPARING LEFT**	2,07%
**MIDAXILLARY INCISION**	0,52%
**RIB EXCISION AND STERNAL ELEVATION**	1,03%

As to the surgical details, differences exist between authors regarding the value of opening the pericardium, and the number and site of the aortopexy stitches. Opening the pericardium allows the stitches to be passed precisely through the aortic wall, while if pericardium is not open, the stitches take the pericardial sac. Surgeons usually pass the stitches via the aortic wall at the origin of the right innominate artery, in one case some additional point on the innominate artery itself is given [15]. Other maneuvers seldom described in association of aortopexy were pexy of the pulmonary trunk to the ductus remnant (according to one report more effective to treat distal left main bronchus malacia than aortopexy itself [18] or the pexy of the tracheal wall.

Whatever approach or technique were used, the efficacy of the aortopexy in treating clinical symptoms of TM was reported in more than 80%, of patients, but there is a significant mortality (6%) and complication rate (16.6% in total), although mostly associated with diagnostic complexity and comorbidity (582 associated comorbidities in 518 patients) of these cases.

These results suggest that aortopexy is an appealing procedure for the treatment of different types of TM, supporting the previous study in a relatively large series of patients performed by our group had shown [6]. The persistence of respiratory infections for many years after aortopexy as reported by three different reports [11,13,14] may suggest that the airway in TM patients remains for some reason prone to this episodes despite the resolution of the respiratory distress and therefore the patients should be followed carefully by their Paediatricians and Respiratory Physicians.

A possible source of complications is represented by the population with TM and GOR. GOR is reported in a significant percentage of patients with OA and TM [10,19]. Both conditions are able to worsen the other, as GOR can cause or maintain a TM by distending the oesophagus or by vagal reflexes and the airway obstruction can predispose to GOR due to the increased work of breathing. In the case of ALTE, both conditions should therefore be investigated and possibly treated. There is no consensus, however, which is the condition to be treated first. In his series [7], Dave performed 6 Nissen fundoplications, in 3 patients before and in 3 after aortopexy and concluded that in his opinion aortopexy should be performed before, in agreement with Kiely [3] but this is influenced by the skills and the preferences of the treating units. Consensus exist that TOF must be ruled out and if necessary treated before aortopexy. Kiely points out that aortopexy can worsen the symptoms by an underlying TOF [3]. In our review, the finding of a recurrent TOF after OA repair was relatively high in TM population undergoing aortopexy [6] and in some cases TOF was diagnosed after aortopexy [3,11]. It is not clear if recurrent TOF might be directly responsible of the TM or if other factors, such as an initial increased distance between the upper pouch and the TOF, could play a role on both TM and risk of recurrence of TOF. Kathami observed that TM was more pronounced when a more aggressive dissection of TOF was performed at the OA repair [20]. It is interesting to observe that TM is much more frequent in association with OA with TOF than with pure OA [7].

A comparative study of different treatments for TM, such as aortopexy, tracheal stenting and others, is beyond the purposes of our study; one of the papers reviewed [21] compared tracheal stenting with aortopexy and concluded that both were effective treatments for TM but that tracheal stents were associated with higher rate of failure, of severe morbidity and mortality. A recent review by Cochrane on the different treatment modalities for TM in children concluded that there are no randomized studies comparing different approaches [1].

## Conclusions

In conclusion, the literature review showed that the aortopexy is effective in the majority of patients with TM, but indications, surgical approach, evaluation of the results varied largely between centres. The main pre-operative investigation is reported as bronchoscopy. The series with a large number of patients are few and randomized trials would be preferable in order to evaluate the different treatment modalities (open or thoracoscopic surgery, endoscopic stent positioning, conservative treatment) if a sufficient number of patients could be included.

## Abbreviations

OA: Oesophageal Atresia; TOF: Tracheo-oesophageal fistula; GOR: Gastro-oesophageal reflux; TM: Tracheomalacia.

## Competing interest

The authors declare that they have no competing interests.

## Authors’ contribution

MT drafted the manuscript, MC helped with the review of the literature and the retrieval of the data from the articles, SS helped to draft the paper and to analyze the data, MJE conceived the study and helped with his experience in conducting the review. All authors read and approved the final manuscript. Contribution of “Cinque per mille dell’IRPEF - Finanziamento della ricerca sanitaria”.

## References

[B1] Masters IB Chang AB Interventions for primary (intrinsic) tracheomalacia in children Cochrane Database Syst Rev 2005 4 CD005304 10.1002/14651858.CD005304.pub2 16235399

[B2] Brawn WJ Huddart SN Tracheoaortopexy via midline sternotomy in tracheomalacia J Pediatr Surg 1991 26 660 662 10.1016/0022-3468(91)90004-D 1941451

[B3] Kiely EM Spitz L Bereton R Management of tracheomalacia by aortopexy Pediatr Surg Int 1987 2 13 15

[B4] Van der Zee DC Bax NM Thoracoscopic tracheoaortopexia for the treatment of life-threatening events in tracheomalacia Surg Endosc 2007 21 2024 2025 10.1007/s00464-007-9250-8 17356936

[B5] Gardella C Girosi D Rossi GA Silvestri M Tomà P Bava G Sacco O Tracheal compression by aberrant innominate artery: clinical presentations in infants and children, indications for surgical correction by aortopexy, and short- and long-term outcome J Pediatr Surg 2010 45 564 573 10.1016/j.jpedsurg.2009.04.028 20223321

[B6] Calkoen EE Gabra HO Roebuck DJ Kiely E Elliott MJ Aortopexy as treatment for tracheo-bronchomalacia in children: an 18-year single-center experience Pediatr Crit Care Med 2011 12 545 551 10.1097/PCC.0b013e3182070f6f 21263370

[B7] Dave S Currie BG The role of aortopexy in severe tracheomalacia J Pediatr Surg 2006 41 533 537 10.1016/j.jpedsurg.2005.11.064 16516630

[B8] Ahel V Banac S Rozmanic V Vukas D Drescik I Ahel V Jr Aortopexy and bronchopexy for the management of severe tracheomalacia and bronchomalacia Pediatr Int 2003 45 104 106 10.1046/j.1442-200X.2003.01660.x 12654081

[B9] Malone PS Kiely EM Role of aortopexy in the management of primary tracheomalacia and tracheobronchomalacia Arch Dis Child 1990 65 438 440 10.1136/adc.65.4.438 2346338 PMC1792197

[B10] Filler RM Messineo A Vinograd I Severe tracheomalacia associated with esophageal atresia: Results of surgical treatment J Pediatr Surg 1992 27 1136 1141 10.1016/0022-3468(92)90575-R 1403550

[B11] Abdel-Rahman U Simon A Ahrens P Heller K Moritz A Fieguth HG Aortopexy in infants and children–long-term follow-up in twenty patients World J Surg 2007 31 2255 2259 10.1007/s00268-007-9221-1 17876663

[B12] Weber TR Keller MS Fiore A Aortic suspension (aortopexy) for severe tracheomalacia in infants and children Am J Surg 2002 184 573 577 10.1016/S0002-9610(02)01054-1 12488172

[B13] Perger L Kim HB Jaksic T Jennings RW Linden BC Thoracoscopic aortopexy for treatment of tracheomalacia in infants and children J Laparoendosc Adv Surg Tech A 2009 19 Suppl 1 249 254 19371150 10.1089/lap.2008.0161.supp

[B14] Vazquez-Jimenez JF Sachweh JS Liakopulos OJ Hügel W Holzki J von Bernuth G Messmer BJ Aortopexy in severe tracheal instability: short-term and long-term outcomes in 29 infants and children Ann Thorac Surg 2001 72 1898 1901 10.1016/S0003-4975(01)03233-7 11789767

[B15] Horvath P Hucin B Hruda J Sulc J Brezovsky P Tuma S Liesler J Skovranek J Intermedite to late results of surgical relief of vascular tracheobronchial compression Eur J Cardiothorac Surg 1992 6 366 371 10.1016/1010-7940(92)90174-V 1497929

[B16] Mok Q Negus S McLaren CA Rajka T Elliott MJ Roebuck DJ McHugh K Computed tomography versus bronchography in the diagnosis and management of tracheobronchomalacia in ventilator dependent infants Arch Dis Child Fetal Neonatal Ed 2005 90 F290 293 10.1136/adc.2004.062604 15857878 PMC1721907

[B17] Schaarschmidt K Kolberg-Schwerdt A Pietsch L Bunke K Thoracoscopic aortopericardiosternopexy for severe tracheomalacia in toddlers J Pediatr Surg 2002 37 1476 1478 10.1053/jpsu.2002.35418 12378458

[B18] Kamata S Usui N Sawai T Nose K Kitayama Y Okuyama H Akira O Pexis of great vessels for patients with tracheobronchomalacia in infancy J Pediatr Surg 2000 35 454 457 10.1016/S0022-3468(00)90213-6 10726688

[B19] Corbally MT Spitz L Kiely E Brereton RJ Drake DP Aortopexy for tracheomalacia in oesophageal anomalies Eur J Pediatr Surg 1993 3 264 266 10.1055/s-2008-1063556 8292576

[B20] Dodge-Kathami A Deanovic D Sacher P Weiss M Gerber AC Clinically relevant tracheomalacia after repair of esophageal atresia: the role of minimal intra-operative dissection and timing for aortopexy Thorac Cardiovasc Surg 2006 54 178 181 16639679 10.1055/s-2005-872954

[B21] Valerie EP Durrant AC Forte V Wales P Chait P Kim PCW A decade of using intraluminal tracheal/bronchial stents in the management of tracheomalacia and/or bronchomalacia: is it better than aortopexy? J Pediatr Surg 2005 40 904 907 10.1016/j.jpedsurg.2005.03.002 15991168

